# 635 PHQ-9 Clinical Assessment: Evidence to Support Post-Injury Psychological Evaluation

**DOI:** 10.1093/jbcr/iraf019.264

**Published:** 2025-04-01

**Authors:** Nicole Kopari, Michael Mosier, Lisa Salvatore

**Affiliations:** UCSF Fresno, Leon S. Peters Burn Center; UCSF Fresno, Leon S. Peters Burn Center; UCSF Fresno, Leon S. Peters Burn Center

## Abstract

**Introduction:**

Burn injury affects both physical and psychological health. While treatment of the physical injury, often gets a lot of attention, roughly 50% of burn survivors report a major depression disorder (MDD). With society’s increasing focus on mental health, we sought to evaluate the presence of depression in adult burn survivors in our outpatient clinic. Identifying a previously unmet need, we sought to increase the use of a standardized screening tool to capture the incidence of MDD and gain administrative support for additional outpatient mental health resources.

**Methods:**

We implemented the PHQ-9 in the outpatient setting of our ABA Verified Burn Center. All burn patients were encouraged to fill out the PHQ-9 form and results were recorded in the electronic medical record (EMR). All results were verified by the clinical team. A referral algorithm was created based on the scoring results in a tiered fashion for clinic follow-up, social work evaluation, or psychology referral. If patients scored >2 on the final question, they were escorted to the emergency department (ED) for immediate evaluation for high risk of suicide.

**Results:**

Since the implementation of the PHQ-9 in April of 2024, a total of 599 patient encounters were reviewed. 206 patients (34%) were female and 393 patients (66%) were male. Females were older with an average age of 46 vs 42.5 and tended to score higher on the pain (4.2 out of 1-10 score) and itch (4.2 out of 1-10 score) scales when compared to the males at 3.7 and 3.3 respectively. 13% of female and 9% of the male respondents self-reported post-traumatic stress or acute stress disorder. Both groups reported poor nightly sleep, with females reporting an average of 5 hours and males an average of 5.3 hours. 41% of females and 32% of males reported returning to their pre-injury activities of daily living. 40% of females and 22% of males reported that their wounds were >95% healed. 36% of females and 27% of males reported a scar yet only 36% of females and males reported wearing compression garments. 29% of females and 20% of males were interested in burn support group activities. Females had higher average PHQ-9 scores of 4.9 compared to 3.9 for males. Based upon our referral algorithm, 39 patients met criteria for psychology referral for severe depression, and 5 patients (over 7 separate patient encounters) met requirement for immediate psychological evaluation in the ED.

**Conclusions:**

With increased focus prioritizing mental health, our burn program sought to better capture the incidence of MDD and identify the need for additional mental health resources in the outpatient setting. By utilizing a validated scoring method, we noted that 38% of our burn patients met the criteria for moderate to severe depression.

**Applicability of Research to Practice:**

Concerned by this significant incidence, we utilized these findings as the basis for advocating to hospital administration for additional mental health support for our burn patients in both the inpatient and outpatient setting.

**Funding for the Study:**

N/A

